# Nurses using physical restraints: Are the accused also the victims? – A study using focus group interviews

**DOI:** 10.1186/1472-6955-6-5

**Published:** 2007-07-17

**Authors:** Claudia KY Lai

**Affiliations:** 1 School of Nursing, The Hong Kong Polytechnic University, Hung Hom, Kowloon, Hong Kong SAR, China

## Abstract

**Background:**

To date, the literature has provided an abundance of evidence on the adverse outcomes of restraint use on patients. Reportedly, nurses are often the personnel who initiate restraint use and attribute its use to ensuring the safety of the restrained and the others. A clinical trial using staff education and administrative input as the key components of a restraint reduction program was conducted in a rehabilitation setting to examine whether there were any significant differences in the prevalence of restraint use pre- and post-intervention. Subsequent to the implementation of the intervention program, focus group interviews were conducted to determine the perspective of the nursing staff on the use of restraints and their opinions of appropriate means to reduce their use.

**Method:**

Registered nurses working in units involved in the study were invited to participate in focus group interviews on a voluntary basis. Twenty-two registered nurses (three males [13.6%] and nineteen females [86.4%]) attended the four sessions. All interviews were audio taped and transcribed verbatim. Other than the author, another member of the project team validated the findings from the data analysis.

**Results:**

Four themes were identified. Participants experienced internal conflicts when applying physical restraints and were ambivalent about their use, but they would use restraints nonetheless, mainly to prevent falls and injuries to patients. They felt that nurse staffing was inadequate and that they were doing the best they could. They experienced pressure from the management level and would have liked better support. Communication among the various stakeholders was a problem. Each party may have a different notion about what constitutes a restraint and how it can be safely used, adding further weight to the burden shouldered by staff.

**Conclusion:**

Studies about restraints and restraint use have mostly focused on nurses' inadequate and often inaccurate knowledge about the use of restraints and its associated adverse effects. These studies, however, fail to note that nurses can also be victims of the system. Restraint use is a complex issue that needs to be understood in relation to the dynamics within an environment.

## Background

The topic of restraint reduction has been under intense scrutiny since the late 1980s, when it began with a public outcry in developed countries arising out of concern with regard to the standard of care in long-term care settings. In Britain, the use of physical restraints on older people is often regarded as abuse [1]. In the United States, a nursing home reform law was enacted in 1987, resulting in an increasing number of studies on restraint use from then onwards. Almost two decades later, however, researchers still find nurses resistant to the notion of removing patients' restraints. Protecting patients from injuries such as falls and preventing treatment disruption are the most important reasons given for the use of physical restraints by all professional groups [2,3]. Reportedly, nurses are most often the personnel who initiate restraint use [4,5].

The attitude of nurses toward restraints is considered one of the main reasons for variations in their use [6,7]. Karlsson and colleagues found that nursing staff with a more positive attitude toward restraint use were more prone to using restraints [8]. Highly educated staff were no more critical of the use of restraints and seclusion than other categories of staff [7]. In a postal questionnaire survey by Lee and colleagues, the consequences of taking off restraints were of concern to the respondents [9]. Their concerns included limited monitoring and poor practice – revealing their ambivalence about restraint use even though they pointed out that the positive side was having brought the situation under control.

In Hong Kong, Chien explored the factors that determine nurses' decisions to apply physical restraints on hospitalized elderly psychiatric patients [10]. Patient safety was found to be a crucial factor accounting for the use of physical restraints in Chien's sample. Staff mostly justified their use of restraints by saying that it was because of the shortage of nurses and facilities, meaning that they could not provide a safe environment for their patients without using restraints. Chien concluded that nurses' attitudes contributed to inappropriate decision-making with regard to restraint use [10].

The common types of physical restraints used in Hong Kong include trunk restraints, limb holders, mitts, lap tables, and bedrails. While most staff choose to use physical restraints [7], the decision itself is often a dilemma [11], and is frequently accompanied by feelings of ambiguity, uneasiness, frustration, and even powerlessness [12]. These feelings could arise from the pressure from management or patients' families, or both. The current study aimed at exploring nurses' views on the use of physical restraints.

## Methods

This study was part of a prospective clinical trial aimed at achieving a reduction in the use of physical restraints in rehabilitation settings. Subsequent to the implementation of a restraint reduction intervention programme, focus group interviews were conducted to explore the perspective of the nursing staff with regard to the use of physical restraints, and their perception of the means available to reduce it. Ethics approval was granted by both the Ethics Review Committee of the university, of which the author is a faculty member, and the institutional review board of the hospital where the study took place. Written information about the study was provided at least two weeks prior to the interviews. Registered nurses working in units involved in the study were invited to participate in focus group interviews on a voluntary basis. Verbal informed consent was obtained from the participants at the beginning of each group session.

Four focus group interviews were conducted between July and August 2004, one in each ward involved in the study. Twenty-two registered nurses (three males [13.6%] and nineteen females [86.4%]) attended the four sessions. The mean number of years of nursing experience was 2.5 (standard deviation [SD] 0.85). The interview took place in the day room next to the unit before the changeover between the morning and evening shifts. All interviews were audio taped and transcribed verbatim. The mean duration of the interviews was 33.2 minutes (SD 5.4).

The semi-structured interview consisted of a discussion around the following questions:

1. what is your opinion of the use of restraints and the hospital's restraint reduction project?

2. what do you think would/could help you reduce the use of restraints in practice?

All focus group sessions were conducted by one member of the research team (the author). She was not involved in the implementation of the interventions. The session was open to all staff and participation was voluntary. The group facilitator and the participants did not know each other. Before each session began, she introduced herself as a member of the project team and explained to the participants the purpose and processes of these meetings. Participants were reassured that although the sessions were audio taped, no names would be recorded. All recordings and transcriptions bore no identifiers that would reveal the identities of the participants. The tapes were transcribed verbatim by a trained research assistant who was present at all of the interviews as an observer. The transcription took place within several days of each interview. Data from the interviews were reviewed for accuracy by the author.

The procedures for analysing the data were adopted from Colaizzi's method [13,14]. They included reading and rereading the transcripts to gain an overall impression of the data. Immersion in the data enables the researcher to develop a sense of the whole of the interview as a context. The words, phrases and sentences were noted to delineate units of participants' meanings. Significant statements, that is, phrases and sentences directly pertaining to the subject matter under investigation, were extracted and grouped into different clusters. The clusters were then read alongside the transcript for the next level of abstraction – delineation into themes to illuminate meanings. To ensure the trustworthiness of the data, first, the theme clusters were re-validated by the researcher by going back to the original transcript using the original statements made by the interviewees. Second, the themes identified were validated by another member of the project team.

## Results

Several themes related to staff members' experience were identified from the transcribed data. These included experiencing internal conflicts, making a strong case against inadequate staffing, and voicing their frustration with the pressure from management. Most of the participants concentrated on talking about their views on the use of restraints rather than specifically on the hospital's restraint reduction project. The themes related to the participants' views on restraint use are discussed below.

### Theme 1: "I will use restraints despite ambivalence and inner turmoil"

Staff were ambivalent about restraint use in practice. They reported not liking to use restraints on patients and yet they felt that they had to apply restraints for various reasons – to prevent treatment disruption, stop self-injury, prevent falls and so on. One participant mentioned:

"I am not worried about having to write an incident report (if a patient fell). Probably we would have to face the family. That is to say, they placed their relative under our care in the hospital, but then we allowed him/her to get hurt. They may put the responsibility on us. That is, they will blame us. It is not as simple as writing a statement. We will feel the burden."

They stated that it was often difficult to judge when to apply a restraint. They felt that it was the dread of the responsibility that steered them toward putting patients in restraints (e.g., RN13-3-1). One member of staff gave a good example to illustrate this guilty feeling when patients fell. A patient who was transferred from another ward did not show any compromised abilities. He was not therefore put in any kind of restraint. Unfortunately the patient later fell in the ward and sustained a hip fracture. The nurse said that luckily the patient's family did not blame the hospital or the nurse. But he said he was upset because his patient was hurt in a fall and he had not done anything (such as applying restraints) to prevent this from happening (RN7-3-1).

Although the participants were ambivalent about using restraints, they said they would use them anyway, "unless they can find something to replace restraints so that we don't need to worry about the responsibility of having a patient fall (RN13-5-2)." This statement by one of the nurses implied that she would continue to use restraints even though she might not feel comfortable doing so.

Nurses in one particular focus group session saw the removal of restraints leading to repeated insertion of, for example, feeding tubes, as causing greater harm to the patient than the restraint itself. They felt that they had applied restraints when there were no other options. One participant said, "...We have spent a lot of time with those old ladies so we know them very well. We know which patients will really become confused ...we try to comply with what is asked of us and take away the restraints...but I must admit that we often have to put the tubes back in...I don't know...because sometimes it can be quite traumatic when you put in a tube." (RN20-6-1)

"But when you insert a Ryle's tube, sometimes you just can't make it in one go. Sometimes you can see that the patient is in tears. And you can't do it even after trying over and over again. If this were a member of my family, I would be very angry because she ends up in such a state...it's so horrible. Sometimes I feel that the patients feel horrible, and as a nurse I feel very bad myself. We know that restraining them with limb holders might make their hands swollen and bruised; I can understand the objection to that. But under certain circumstances, is it really to their benefit when the restraints are removed? This is what actually happens." (RN20-6-3)

Not using restraints gave staff an uneasy feeling, whereas using restraints apparently provided a certain sense of security for the nurses. "...Would we consider having the bedrail up on one side, with the other side down and just positioning the patient with pillows? But then we would feel it was unsafe... We would be so worried." (RN18-2-1)

Staff were nevertheless aware of the potential dangers of restraint use (e.g., RN8-3-2). "Sometimes we find it so funny. Even with the lap table on, and with an anti-slip seating mat, the patient can still slip onto the ground." (RN12-8-1) The participant was aware that this scenario she described could have led to injury or suffocation by entrapment.

### Theme 2: "We are always short-staffed at work and we are just trying to do the best we can"

The lack of nurses to support a reduction in restraint use was mentioned by nearly all of the participants. They said that the low staffing level led them to apply restraints so as to "reduce" the number of accidents. Some suggested that there should be an extra pair of hands to reinsert feeding tubes if they were pulled out by patients (e.g., RN10-4-2). They would also like to see at least one staff member working in each cubicle (e.g., RN10-5-11).

"In fact, really... we feel that there is nothing much we can do...in many situations, one member of staff has to look after two and a half to three cubicles; one cubicle has eight beds, and there is one nurse and one health care aide looking after five cubicles of patients. Well, each of us has our work to do. Both the HCA and I have the responsibility to prevent falls. For the sake of patients' safety, well, we have to prevent them from falling, so we have to do everything we can." (RN8-6-1)

"Both A and P shifts had only two nurses. One nurse would have to be responsible for 30 plus patients, assisted by one health care aide." (RN18-4-1) "Well, I think to have more staff is the most basic need." (RN17-3-1) "Or the other suggestion we would make is to have fewer patients." (RN14-3-2) The participants believed that they had already done their best.

### Theme 3: Frustrated as a result of pressure from management and the need to conform

Nursing staff were frustrated by the pressure they experienced in implementing restraint reduction policies. This sense of frustration was strong. A hospital policy with regard to fall prevention had recently been put into operation. The goal was to ask staff to reduce the fall rate by 10%, and the staff indicated that they felt pressured.

The participants complained that the volume of work subsequent to a fall also added to the stress they already felt. One of them was articulate in describing the situation.

"In fact the management is very important...For example, when the ward manager comes around the unit and asks us to take off the restraints, even if we feel inside that this one cannot do without a restraint, we still need to try...The work that you have to do after a patient falls is considerable. I have roughly estimated that, even if it is only a minor fall, the work that follows takes at least an extra hour...you have to get the patient up, reassure him, get him back into bed, do your obs (observations), that is to say, to ask them about what happened. Then, if the patient needs treatment, you call the doctor; probably you will have to arrange for the patient to have an x-ray, and then you will have to report it – you will have to write up a statement, update the patient's record... and then you will have to inform the family, etc. etc. Roughly you would need an hour." (RN8-3-3)

When the participants were asked why they would feel responsible even when it was not necessarily their fault, they said that it was because they were held accountable. One participant recounted a personal experience with the problem of writing up a statement concerning a fall incident. It put a lot of pressure on her, affected her sleep, and made her cry. "I didn't want her to fall. I was working in another cubicle. I had no idea..." She was upset because she had had to rewrite this statement over and over again until the management was satisfied with it (RN18-7-2). Participants felt responsible because they knew that eventually the front line staff would be blamed. "...No matter what, no matter if you are due to go off work, you still need to write this statement." (RN18-8-1)

One participant mentioned that applying restraints was not an individual's choice, but rather the overall climate of the unit had a role to play. The culture here made them worry about having a patient fall.

"...We are different here. The culture we have here means that if a patient falls, we are very worried about the matter of responsibility. Even our senior management is very concerned. And so it makes us nervous. Because we have to be accountable, we will first try to use restraints. I feel that this is one of the reasons why the restraint rate is high for us here." (RN13-3-5) They felt that one way the management could help them would be to streamline the reporting procedure (e.g., RN8-4-3).

### Theme 4: Communication problems among various parties, each with a different notion of what function(s) a restraint could serve, did not facilitate restraint reduction

Insufficient communication between different parties – management, patients, patients' families, and front line staff was given as a reason for staff resorting to restraint use. The importance of communication with families at all times was stressed repeatedly by the participants. They felt that families' attitudes and misunderstandings about the use of physical restraints added to the pressure already on them. The expectation or sometimes demand from families to ensure that nothing untoward would happen to the patients was unreasonable to them. "Sometimes it means that the family makes us restrain (the patient)." (RN14-1-2)

"...They (families) would say it is OK both ways – either to restrain or not to restrain. But if the patient really fell, what would happen then? What if the patient got a fracture?" (RN11-4-3)

Reportedly, families and patients differed in their attitudes. "...There are some patients who request restraints. For example, like some rehab patients, they don't feel secure if you lower their bed rails. One patient would say, 'Nurse, you better put them up for me. I fear that I may fall...' Even if you tell them that if they are careful, it will be fine with just one bedrail, they strongly request that you put up both sides. 'If I need to get out of bed, I will press the call bell and ask you to lower them for me."' (RN18-1-1)

One family asked one of the participants to tie up even the head of a patient. "I asked why they would ask that, and she told me that the head moved too much. So I told the family we can't do that." (RN 14-3-1)

Even if families raised objections to the use of restraints, the nursing staff would try to convince them to "buy into" their view.

"Usually we try to convince the family rather than take away the restraint. We explain to the families what are the uses of the restraint, that we use them just in case, or that the lap table can help the patient eat his meal sitting up and so on. Usually we are able to convince the family rather than taking away the restraint."

The respondents' found the viewpoints of different stakeholders conflicting. The lack of communication among various parties involved rendered it difficult for them to follow through what they thought would be best for patients. Consequently, they resorted to the usual practice of applying restraints.

In summary, the hospital itself had a newly instituted "fall reduction programme" in place which made the RNs feel pressured. They believed that the fall rate would increase if restraints were removed. They also thought that patients would feel insecure without restraints. The majority of the participants seemed to be quite pessimistic, believing that they had already done whatever they could and that nothing else could change the status quo except better staffing. Most of the participants were reluctant to accept that they had to reduce the use of restraints, and were negative toward the idea. They felt that it was unreasonable to talk about restraint reduction without increasing the number of nurses. A general sentiment of the participants was that the management did not have much in place to support staff but rather created a lot of hassles when patients fell.

## Discussion

The overriding theme identified by the participants was that to prevent falls was nurses' responsibility and the use of physical restraints a means to enable nurses to shoulder this responsibility. Similar to the findings reported in Wynn's questionnaire-based survey [7], a majority of the participants in this study believed that they had used restraints correctly and that nothing more could have been done to facilitate restraint reduction except better staffing. Nurses consistently cited insufficient staffing as one of the major reasons for restraint use [15,16].

During the focus group sessions, two participants stated that there were only two nurses for both the morning and evening shifts, and that one nurse, assisted by one health care aide, would have to be responsible for over 30 patients. Their statements were corroborated by other participants who were present at the same time. Subsequent verifications with the management of the hospital came up with somewhat different figures. According to the management, there were eight nursing staff (including nursing officers and staff nurses [registered and enrolled nurses]) and four health care aides for a 64-bed ward for both the morning and evening shifts. The accuracy of these different sets of figures is beside the point for the purpose of this paper. The key message from the participants was that they were overburdened with patient care responsibilities and that they were highly stressed and under-staffed.

With regard to the relationship between staffing and restraints, the literature, however, indicates otherwise. Magee and colleagues noted that better staffing was paradoxically related to an increase in the use of restraints [17]. Others reported that nurses appeared to restrain patients for their own convenience but used low staffing levels as an excuse to justify their decisions [e.g. [18]]. A study in Japan examined the relationship between physical restraints in nursing homes and their institutional characteristics, and found that those characteristics which correlated with the use of physical restraints were intensive staffing, holding periodical care conferences, and having unit care [19].

Although many may suggest that the decision to use or remove physical restraints falls mainly under the professional authority of nurses and physicians [20], it needs to be mentioned that in a dynamic health care environment such as that of a hospital, many forces are at work [8]. The voice of the participants as illuminated in this study shows us the tension they feel and the inner turmoil they experience. Because of the continuous interactions among patients, systems, and care giving staff, organizational characteristics play an important role in the use of physical restraints [21]. If fundamental changes in practice were what the profession or institution pursued, a lot more thought and resources would be needed in order to realize desirable outcomes. In order to achieve restraint reduction, the factors affecting clinical decision making must be dealt with at the facility level [22]. The research direction of examining the use of restraints with institutional characteristics [e.g. [19]] is probably a useful ways to help us uncover the complexities of the problem with restraint use.

Most staff members preferred the use of physical restraints although they were aware that it was not favourable to patients. Many of them seemed quite adamant in their beliefs and were convinced that in view of the circumstances they were in, there was no better way out. Nearly all nurses in this study expressed varying degrees of discomfort associated with restraint use but at the same time referred to it as their duty to protect the patient from harm and injury. This could indicate that participants were attempting to balance the scale between conflicting values in their decision-making. Strumpf and Evans (1988) interviewed 20 elderly restrained patients and their primary nurses. They reported that the decision to restrain created a conflict among the nurses between the need to protect and their professional values. To date, the struggle nurses feel in caring for patients continues after almost two decades [23].

In spite of the internal struggle, the participants would still use restraints, believing that their use would prevent falls and other fall-related problems. As Evans and Strumpf emphasized, "myths are powerful determinants of behavior, even in professional practice" [24]. Letizia and her team state that fear shapes our attitude and renders changes to practice difficult [25]. In order to move forward, nurses as individuals must carefully reflect on our practice and re-appraise our stance to counteract these beliefs. Health care facilities as institutions also need to be accountable for the institutional norms and culture being created, and be willing to modify or change them, even though changing them may imply a lot more work than simply providing staff education.

Broadly speaking, the perspective adopted by researchers as reported in the literature includes nurses with inadequate knowledge about restraint reduction or restraint alternatives [e.g. [10,26,27]] and nurses who opt to maintain their own sense of comfort and security over the autonomy and dignity of patients [e.g. [28]]. Nurses seem to have been identified as the crux of the problem in restraint use. However, it needs to be understood that nursing staff may be victims in the system. More often than not, discussions in the literature fail to note that management might be one of the possible obstacles to change.

The participants put forth a strong case as to how management could have impeded instead of facilitating a change in practice with too much bureaucracy in place, and with a stance that placed great emphasis on the consequences of falls. Rader and colleagues pointed out that nursing home staff would not and could not change unless they had administrative support [29]. Administrative support is essential for restraint reduction [30] and must be "visible and unambiguous" [31].

It needs to be emphasized that this was only a one-site study, rendering generalization of the findings difficult. It is high time we investigated further the complexity of issues surrounding restraint use from a qualitative perspective, and identified solutions that would address the total situation. Many large scale empirical studies have adopted a quantitative approach to investigating the phenomenon. For instance, data was captured from minimal data sets collected in nursing homes to identify factors predictive of, or associated with, restraint use. More in-depth studies to understand the dynamics of restraint use within the health care system are required. For instance, non-participatory observational studies may help to shed light on the contexts in which restraints are being applied. The kinds and level of perceived stress of nurses associated with the application or removal of restraints will help researchers to better understand the perspective of nurses – why and how they use restraints on patients. The use of the case study method, which is not now in vogue in studying restraint use, can in fact provide insights into the dynamics between different parties involved in the process of deciding to restrain the patient, and the nurses' role in it. It is also important to examine nurses' attitude and behaviour when they feel that they are not empowered, because nursing actions impact on patient outcomes.

## Conclusion

The participants were more interested in discussing their views and experiences in the use of restraints than the restraint reduction project conducted in their hospital, highly likely related to the strong sentiments they felt toward restraint use. They expressed dissatisfaction with management with regard to the pressure they felt was placed on them to reduce fall rates and restraint use. Most participants thought that they had already done their best, and emphasized that better communication among all stake holders was significant in resolving the use of restraints.

Not all falls are preventable, and evidence shows that restraint use will not prevent falls. A restraint-free environment will not materialize until staff, family, and administrators view restraints as the problem and not the solution [32]. Nurses should not be regarded as the main source of the problem but a potential solution. In fact, the phenomenon of restraint reduction should be viewed from a more macroscopic perspective. This study calls for a new angle in studying the complex dynamics of restraint reduction, and will hopefully start a discourse on how best to move restraint reduction studies forward.

## Competing interests

The author(s) declare that they have no competing interests.

## Pre-publication history

The pre-publication history for this paper can be accessed here:


